# Endovascular treatment for acute basilar artery occlusion: a single center retrospective observational study

**DOI:** 10.1186/s12883-019-1551-8

**Published:** 2019-12-06

**Authors:** Xuan Sun, Xu Tong, Feng Gao, Huiting Lao, Zhongrong Miao

**Affiliations:** 1 Department of Interventional Neurology, Beijing Tiantan Hospital, Capital Medical University, 119 South Fourth Ring West Road, Fengtai District, Beijing, China; 2 Department of Medicine, Queen Elizabeth Hospital, Hong Kong, China

**Keywords:** Acute ischemic stroke, Basilar artery occlusion, Endovascular treatment

## Abstract

**Background:**

Endovascular treatment (EVT) is now considered the gold standard for select patient populations with anterior circulation stroke; however, data on the treatment of posterior circulation stroke are less clear. This study aims to determine the characteristics and treatment outcomes of patients with acute basilar artery occlusion (BAO) and to evaluate the effectiveness and safety of EVT for patients with acute BAO in a high-volume stroke center.

**Methods:**

This study included 187 consecutive patients with acute BAO who underwent EVT from January 2012 to July 2018 in the Beijing Tiantan Hospital. The baseline characteristics, procedure parameters, and functional outcome were assessed.

**Results:**

Among the 187 patients, 138 (73.8%) underwent mechanical thrombectomy with a stent retriever, 33 (17.6%) underwent direct intracranial angioplasty (balloon dilation and/or stent implantation) for underlying severe intracranial atherosclerotic disease, and 91 (48.7%) underwent combined mechanical thrombectomy and angioplasty. Successful recanalization [modified Thrombolysis in Cerebral Infarction (mTICI) grade 2b-3] was achieved in 158 patients (84.5%). Overall, the rates of functional independence [modified Rankin Scale (mRS) 0–2] and favorable outcome (mRS 0–3) at 90 days were 36.4 and 49.2%, respectively, and 90-day all-cause mortality was 20.3%.

**Conclusion:**

EVT was effective and safe for treating patients with acute BAO.

## Background

Acute ischemic stroke (AIS) caused by basilar artery occlusion (BAO) has devastating effects on patients and has high morbidity and mortality rates. Among patients treated conventionally with antiplatelets or anticoagulation, the rate of poor outcomes remains high (80%) despite recent advances in stroke care [1, 2]. In the registry of Basilar Artery International Cooperation Study (BASICS) [1], the proportion of favorable outcomes [modified Rankin Scale (mRS) 0–3] in the antithrombotic treatment group was 21.9, 33.9% in the intravenous thrombolysis (IVT) group, and 17.5% in the intra-arterial treatment (local thrombolysis) group. The mortality rates in the groups were 30.6, 33.9, and 40.6%, respectively. However, new-generation endovascular treatment (EVT) devices such as “Solitaire” and “Trevo” stent retrievers were not used in the BASICS [3, 4].

Modern EVT techniques, particularly mechanical thrombectomy (MT) with stent retrievers, have been validated by several large randomized controlled trials [5, 6]. Recently, more studies embracing the New England Center Posterior Circulation Registry have shown no significant differences in the clinical features of posterior circulation stroke (PCS) compared with those with anterior circulation stroke (ACS) and a more benign outcome in patients with PCS [7, 8]. The efficacy and safety of MT in patients with acute Bao are uncertain due to the lack of case series and case reports showing the efficacy and safety of posterior circulation thrombectomy in a single-arm study. Our intention was to depict our experience with EVT for acute BAO at a high-volume stroke center in China and to assess its feasibility and safety.

## Methods

### Patient selection

We performed a retrospective analysis of consecutive patients presenting with BAO treated by EVT in Beijing Tiantan Hospital from January 2012 to July 2018. IVT with tissue plasminogen activator (tPA) was acceptable before EVT, consistent with current guidelines [9]. The protocol was approved by the Institutional Review Board of Beijing Tiantan Hospital.

Plain computed tomography (CT) of the brain was performed first, followed by either computed tomography angiography (CTA) or magnetic resonance imaging (MRI) with magnetic resonance angiography (MRA) to confirm the presence of acute BAO and to exclude both large brainstem infarction and intracranial bleeding. Imaging modality selection relied on physician preferences and the logistics of scheduling the procedures. We retrospectively analyzed clinical and radiologic data of these patients, including age; sex; stroke risk factors including hypertension, diabetes mellitus, hypercholesterolemia, and current smoking; initial stroke severity as expressed by the National Institutes of Health Stroke Scale (NIHSS) score; initial imaging modality; prior use of IVT; type of EVT devices; onset to puncture time; procedure time; onset to recanalization time; collateral status; and etiology of stroke according to the Trial of Org 10,172 in Acute Stroke Treatment (TOAST) classification [10]. The modality of treatment was a joint decision between the neurointerventionalist and the stroke team taking care of the patient.

### Endovascular treatment

All EVT procedures were performed by a neurointerventionalist with more than 50 cases of experience in neurovascular intervention in MT for AIS. Cerebral angiography and EVT were performed under general anesthesia or conscious sedation after evaluation by a dedicated anesthesiology team. The thrombectomy technique was chosen at the interventionalist’s discretion, using either a stent retriever or aspiration first, with a possible switch to another strategy in case of recanalization failure [modified Thrombolysis in Cerebral Infarction (mTICI) grade 0-2a] with the first approach. If underlying intracranial atherosclerotic stenosis (ICAS) was revealed, intracranial angioplasty or stenting was performed when needed. If patients who were antiplatelet-naive undergoing angioplasty and/or stenting received an loading dose of aspirin (300 mg) and clopidogrel (300 mg) orally or via a nasogastric tube immediately after the intervention. During operative bridging with intravenous IIb/IIIa inhibitor was also allowed at the operator’s discretion. Dual antiplatelet therapy was preserved for at least 3 months, followed by aspirin or clopidogrel for life.

### Image interpretation

Pretreatment neurovascular images, including posterior circulation Acute Stroke Prognosis Early CT Score (pc-ASPECTS), Pons-Midbrain Index based on diffusion-weighted imaging, and two previous collateral scales [posterior communicating artery (PCoa) and American Society of Interventional and Therapeutic Neuroradiology/Society of Interventional Radiology (ASITN/SIR)] [11, 12] described in the setting of BAO, were retrospectively interpreted by two independent trained neurointerventionalists blinded to clinical data. A third neurointeventionalist with 20 years’ experience was involved to resolve any disputes. All collateral scales mentioned above were evaluated by digital subtraction angiography.

### Outcome measurement

Primary effectiveness outcome measures were functional independence and favorable outcome at 90 days after the procedure. Functional independence was defined as an mRS score of ≤2. Favorable outcome was defined as an mRS score of ≤3, in accordance with the BASICS definition [1]. The mRS was assessed by blinded trained interviewers over the phone using a standardized interview protocol. Other effectiveness outcome measures included the rates of successful recanalization (mTICI 2b-3) and complete recanalization (mTICI = 3) [13].

Safety outcome measures were 90-day mortality and rates of intracerebral hemorrhage and symptomatic intracerebral hemorrhage (SICH) within 24 h. SICH was defined on the basis of the European Cooperative Acute Stroke Study III (ECASS III) criteria [14].

### Statistical analysis

Study data were collected on standard forms, evaluated for completeness, and double keyed into an EpiData statistics data document. Baseline and outcome data were described using means (standard deviations) and/or medians (25th and 75th percentiles) for continuous variables. Frequencies and/or proportions were used for categorical variables. Pearson’s chi-square test or Fisher’s exact test was used to compare the differences in frequencies and/or proportions. All tests were two-tailed, and statistical significance was determined at an α level of 0.05. All statistical analyses were performed with the statistical software packages R (http://www.R-project.org, The R Foundation) and Empowerstats (http://www.empowerstats.com, X&Y Solutions, Inc., Boston, MA).

Data availability statement: All data generated or analyzed during this study are included in this published article [and its Additional file 1].

## Results

A total of 187 patients with BAO underwent EVT during the study period. Their mean age was 60 ± 10 years, with a median admission NIHSS score of 22 [interquartile range (IQR) 10–34]. Baseline characteristics and procedure and outcome parameters are summarized in Tables 1 and 2. The median time from BAO onset to puncture was 7 h (IQR 5–10). In terms of procedure parameters, stent retrievers were more frequently used (73.8%) than intra-arterial thrombolysis (23.0%) or stenting (49.7%). General anesthesia was used in 78.6% of the cases, and the median procedure length was 1.5 h (IQR 1–2). Successful recanalization (mTICI 2b-3) and complete recanalization (mTICI 3) were achieved in 158 (84.5%) and 126 (67.4%) patients, respectively. Intracerebral hemorrhage occurred in 29 (15.5%) patients. SICH within 24 h occurred in 8 (4.3%) patients. Ninety-day functional independence and favorable outcome were achieved in 68 (36.4%) and 92 (49.2%) patients, respectively, while mortality at 90 days was 20.3% (Table 2).

**Table 1:** Values are numbers with percentages in parentheses, unless indicated otherwise

Variable name	Overall cohort (*n* = 187)
Demographic data	
Age, mean (SD), years	60 (10)
Male sex	157 (84.0)
Vascular risk factors	
Hypertension	133 (71.1)
Diabetes mellitus	51 (27.3)
Dyslipidemia	30 (16.0)
Coronary heart disease	21 (11.2)
Atrial fibrillation	11 (5.9)
Prior stroke	37 (19.8)
Premorbid mRS ≥ 3	9 (4.8)
Current smoking	70 (37.4)
Clinical characteristics	
SBP, mean (SD), mmHg	160 (25)
NIHSS score, median (IQR)	22 (10–34)
GCS score, median (IQR)	8 (3–13)
pc-ASPECTS on DWI, median (IQR)	6 (5–8)
PMI on DWI, median (IQR)	2 (1–4)
Occlusion site	
Proximal BA (including intracranial VA)	104 (55.6)
Middle BA	54 (28.9)
Distal BA	29 (15.5)
Tandem lesion	25 (13.4)
Underlying ICAS	117 (62.6)
Presence of PcomA	
No	68 (36.4)
Unilateral	88 (47.1)
Bilateral	31 (16.6)
ASITN/SIR collateral system	
Grade 0–1	78 (41.7)
Grade 2	87 (46.5)
Grade 3–4	22 (11.8)
Stroke subtype by TOAST criteria	
Large artery arteriosclerosis	151 (80.7)
Cardioembolic	29 (15.5)
Other or unknown etiology	7 (3.7)

Abbreviations: *BA* basilar artery, *DWI* diffusion weighted imaging, *GCS* Glasgow Coma Scale, *ICAS* intracranial atherosclerotic stenosis, *IQR* interquartile range, *mRS* modified Rankin Scale, *NIHSS* National Institutes of Health Stroke Scale, *pc-ASPECTS* posterior circulation Acute Stroke Prognosis Early CT Score, *PMI* Pons-Midbrain Index, *SBP* systolic blood pressure, *SD* standard deviation, *TOAST* Trial of Org 10,172 in Acute Stroke Treatment, *VA* vertebral artery

**Table 2:** Values are numbers with percentages in parentheses, unless indicated otherwise

Variable name	Overall cohort (n = 187)
Procedural features	
Prior use of intravenous tPA	36 (19.3)
General anaesthesia	147 (78.6)
Use of Solitaire retriever	138 (73.8)
No. of passes	
≤ 1	70 (50.7)
2	38 (27.5)
≥ 3	30 (21.7)
-arterial tPA or Urokinase	43 (23.0)
Balloon angioplasty	101 (54.0)
Stenting	93 (49.7)
Onset to puncture time, median (IQR), hours	7 (5–10)
Procedure time, median (IQR), hours	1.5 (1–2)
Onset to recanalization time, median (IQR), hours	8.5 (6–11)
Procedural complications	
Embolization in distal or new territory	18 (9.6)
Arterial dissection	6 (3.2)
Arterial perforation	8 (4.3)
Target-vessel reocclusion	17 (9.1)
Outcome measures	
Successful recanalization (mTICI 2b-3)	158 (84.5)
Complete recanalization (mTICI 3)	126 (67.4)
Hemorrhagic transformation within 24 h	29 (15.5)
SICH (ECASS III definition) within 24 h	8 (4.3)
Functional independence (mRS 0–2) at 90 days	68 (36.4)
Favorable outcome (mRS 0–3) at 90 days	92 (49.2)
Mortality at 90 days	38 (20.3)

Abbreviations: *ECASS* European Cooperative Acute Stroke Study, *IQR* interquartile range, *mRS* modified Rankin Scale, *mTICI* modified Thrombolysis in Cerebral Infarction, *SICH* symptomatic intracerebral hemorrhage, *tPA* tissue Plasminogen Activator

**Fig. 1:**
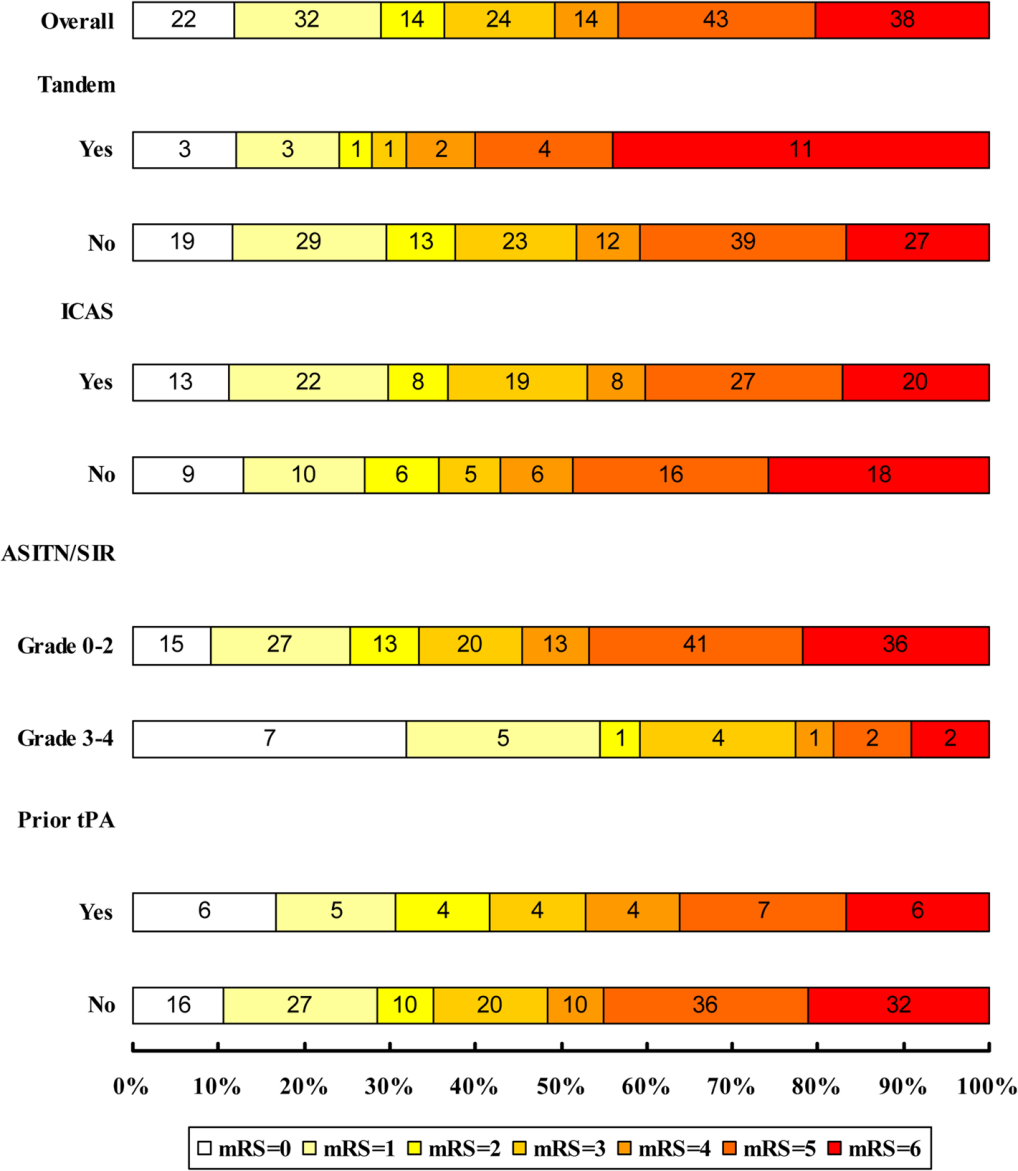
The Distribution of 90-Day mRS in ABAO Patients with Different Subgroup

**Fig. 2:**
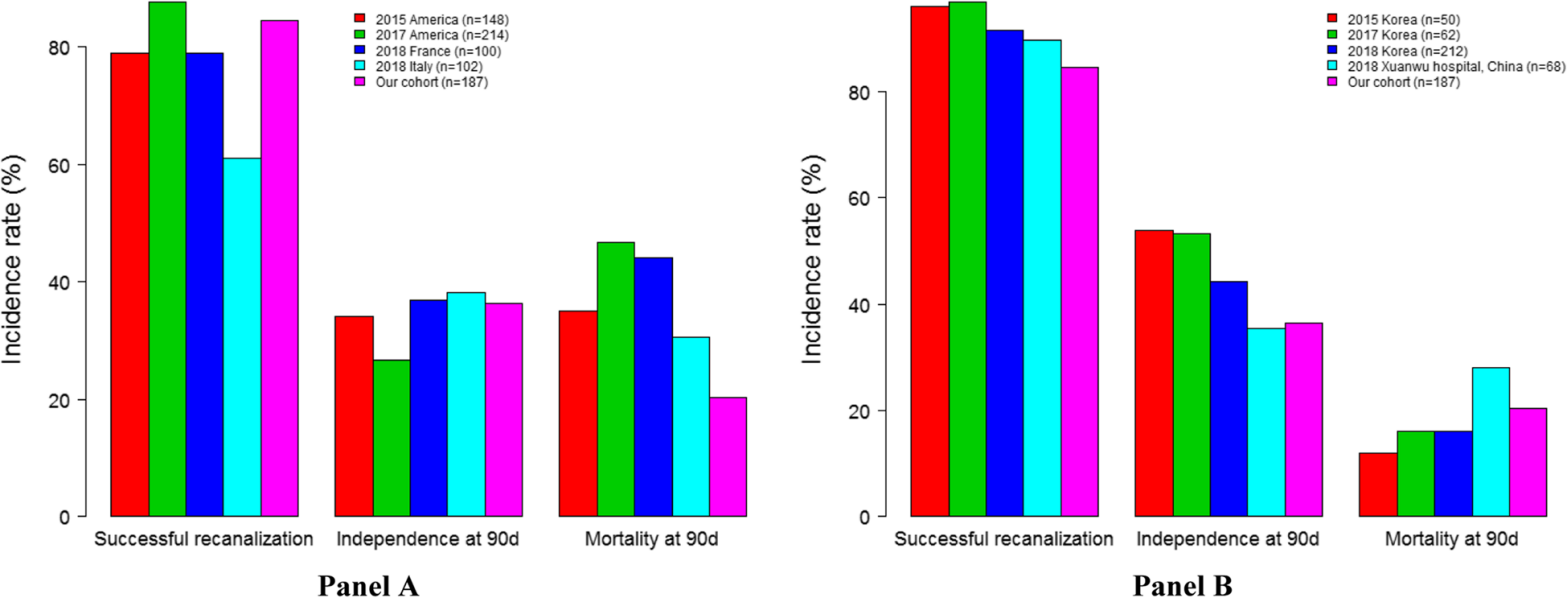
The clinical outcome compared with other Asian and western studies

Results from subgroup analysis in patients with acute BAO are shown in Table 3 and Fig. 1. Of the 187 patients, 117 (62.6%) had underlying ICAS at the occlusion site. Patients with ICAS and those without ICAS showed similar clinical outcomes. There were no significant differences between the two groups in successful recanalization rate (85.5% versus 82.9%; *P* = 0.63), SICH within 24 h (3.4% versus 5.7%; *P* = 0.48), functional independence (36.8% versus 35.7%; *P* = 0.89), favorable outcome (53% versus 42.9%; *P* = 0.18) and mortality (17.1% versus 25.7%; *P* = 0.16) at 90 days. Compared with other patients, patients with tandem vertebrobasilar occlusions appeared as a possible predictor of mortality (44.0% versus 16.7%, *P* = 0.01) and had a lower recanalization rate (60.0% versus 88.3%, *P* <  0.01). No differences in effectiveness and safety outcomes were observed in patients with or without prior use of intravenous tPA (*P* >  0.05). ASITN/SIR collateral scores ≥3 were associated with functional independence (59.1% versus 33.3%, *P* = 0.03) and favorable outcome (77.3% versus 45.5%, *P* = 0.01). Our study demonstrated no difference in successful recanalization, functional independence, and mortality, comparable with results of previous studies from Asian countries. However, our study showed a lower mortality at 90 days compared with that in previous studies from Western countries (Fig. 2).

**Table 3:** Subgroup Analysis of Clinical Outcomes in ABAO Patients

Outcome variables	Subgroups											
	Tandem lesion			ICAS			ASITN/SIR collateral system			Prior use of intravenous tPA		
	Yes (*n* = 25)	No (*n* = 162)	*P* value	Yes (*n* = 117)	No (*n* = 70)	*P* value	Grade 0–2 (*n* = 165)	Grade 3–4 (*n* = 22)	*P* value	Yes (*n* = 36)	No (*n* = 151)	*P* value
Successful recanalization (mTICI 2b-3)	15 (60.0)	143 (88.3)	< 0.01	100 (85.5)	58 (82.9)	0.63	137 (83.0)	21 (95.5)	0.21	30 (83.3)	128 (84.8)	0.80
Complete recanalization (mTICI 3)	13 (52.0)	113 (69.8)	0.11	77 (65.8)	49 (70.0)	0.55	108 (65.5)	18 (81.8)	0.15	26 (72.2)	100 (66.2)	0.49
Hemorrhagic transformation within 24 h	4 (16.0)	25 (15.4)	> 0.99	16 (13.7)	13 (18.6)	0.37	26 (15.8)	3 (13.6)	> 0.99	7 (19.4)	22 (14.6)	0.45
SICH (ECASS III definition) within 24 h	2 (8.0)	6 (3.7)	0.29	4 (3.4)	4 (5.7)	0.48	7 (4.2)	1 (4.6)	> 0.99	1 (2.8)	7 (4.6)	> 0.99
Functional independence (mRS 0–2) at 90 days	7 (28.0)	61 (37.7)	0.38	43 (36.8)	25 (35.7)	0.89	55 (33.3)	13 (59.1)	0.03	15 (41.7)	53 (35.1)	0.46
Favorable outcome (mRS 0–3) at 90 days	8 (32.0)	84 (51.9)	0.07	62 (53.0)	30 (42.9)	0.18	75 (45.5)	17 (77.3)	0.01	19 (52.8)	73 (48.3)	0.63
Mortality at 90 days	11 (44.0)	27 (16.7)	0.01	20 (17.1)	18 (25.7)	0.16	36 (21.8)	2 (9.1)	0.26	6 (16.7)	32 (21.2)	0.65

Abbreviations: *ASITN/SIR* American Society of Interventional and Therapeutic Neuroradiology/Society of Interventional Radiology, *ECASS* European Cooperative Acute Stroke Study, *ICAS* intracranial atherosclerotic stenosis, *mRS* modified Rankin Scale, *mTICI* modified Thrombolysis in Cerebral Infarction, *SICH* symptomatic intracerebral hemorrhage, *tPA* tissue Plasminogen Activator

## Discussion

We analyzed the outcome of EVT in patients with BAO at our institution over the past 5 years. In total, 36.4% of the patients had functional independence (mRS 0–2) and 49.2% had a favorable outcome (mRS 0–3) at 90 days, which were comparable with results of the recently published HERMES meta-analysis of EVT for anterior circulation stroke (46% versus 56.9%) [5]. Similar findings were also reported in a recent study on 436 patients with acute large vessel occlusion of anterior and posterior circulation treated with EVT [15]. Our findings demonstrate that when patients were carefully selected and appropriately treated with EVT, those with posterior large vessel occlusions can achieve comparable safety and efficacy to those with anterior large vessel occlusions. The patients with BAO in our cohort achieved a similar clinical outcome to those in other Asian studies [16–19]. However, they had a significantly lower mortality (about 20%) than those in studies by Western researchers [12, 20–22], although the enrolled patients in our study had a relatively higher NIHSS score (media*n* = 22). This could be explained by the single-center effect attributable to developed interventional techniques and standardized post-operative management. Improving the technology and management of EVT may lead to better recanalization and subsequently to better outcomes. This may also explain why our study had a higher rate of favorable outcomes than previously published studies [23, 24].

Our study shows four major findings: First, EVT combined with IVT for BAO failed to show superior outcomes when compared to EVT alone. The issue of whether prior IVT confers a benefit over direct MT alone has not been settled even for patients with anterior circulation, although studies are underway [25]. Second, tandem vertebrobasilar occlusions were associated with poor outcome and higher mortality. However, a previous study showed that patients with tandem lesions who were comparable with those without tandem vertebrobasilar occlusions presented similar clinical outcomes [26]. Only in recent years has occlusion of the vertebral artery origin been more fully characterized and defined as a potential source of embolic stroke and as a potential predictor of mortality in acute BAO [27]. Third, rescue treatments such as angioplasty with or without stenting and intra-arterial infusion of antiplatelet drugs usually needed in the target artery with underlying ICAS which can make the thrombectomy procedure more complicated [28]. ICAS is an important cause of stent-retriever thrombectomy failure, particularly in Asian patients [29, 30]. In our study, 117 patients had underlying ICAS, and 75 underwent intracranial angioplasty (balloon dilation and/or stent implantation) after first-line MT. We achieved similar rates of successful recanalization (85.5% versus 82.9%), functional independence (36.8% versus 35.7%), favorable outcome (53.0% versus 42.9%), SICH (3.4% versus 5.7%), and mortality (17.1% versus 25.7%) to those observed in patients without ICAS. Our study suggests that such rescue treatments are effective and safe for treating underlying ICAS in patients with acute BAO. Finally, collateral circulation has been shown to affect recanalization, haemorrhagic transformation, and clinical outcomes in patients with anterior circulation stroke [31–33]. In this series, we used the ASITN/SIR and PCoA scales to assess the collateral status and found a significant correlation between ASITN/SIR and 90-day outcomes, but PCoA collaterals were not significantly associated with clinical outcome.

Our study has several limitations. It was a retrospective, non-randomized controlled study. Data were obtained from a single high-volume stroke center with an interventionalist highly experienced in performing the procedure, so the results may not be replicable in other lower-volume stroke centers. However, the strength of our study is that it represents one of the largest cohorts of patients with BAO treated with EVT. The broad inclusion criteria from stroke severity and time of presentation and variability in procedure parameters allow us to improve our understanding of the specific needs of this patient population and appropriate therapeutic strategies. Future multicenter studies are needed to validate our findings.

## Conclusions

our study indicates that EVT for patients with BAO should be reasonable in the modern era despite lack of data from randomized clinical trials to establish the best treatment for BAO.

## Supplementary information

**Additional file 1:** s1:Comparistions of patients' charactristics between outcomes (mRS 3-6 vs. mRS 0-2). s2:Comparistions of patients' charactristics between 90d alive vs. 90d death. s3:Comparistions of patients' charactristics between NO HT within 24h vs. HT within 24h. s4:Comparistions of patients' charactristics between poor clooateral vs. good collateral

## Data Availability

The datasets used and/or analysed during the current study available from the corresponding author on reasonable request.

## Abbreviations

ACS: Anterior circulation stroke
AIS: Acute ischemic stroke
BAO: Basilar artery occlusion
CT: Computed tomography
CTA: Computed tomography angiography
EVT: Endovascular treatment
EVT: Endovascular treatment
ICAS: Intracranial atherosclerotic stenosis
IQR: Interquartile range
IVT: Intravenous thrombolysis
MRA: Magnetic resonance angiography
MRI: Magnetic resonance imaging
mRS: Modified Rankin Scale
MT: Mechanical thrombectomy
mTICI: Modified Thrombolysis in Cerebral Infarction
NIHSS: National Institutes of Health Stroke Scale
pc-ASPECTS: Prognosis Early CT Score
PCS: Posterior circulation stroke
SICH: Symptomatic intracerebral hemorrhage
tPA: Tissue plasminogen activator
