# Supplementary material for: Endovascular treatment for acute basilar artery occlusion: a single center retrospective observational study
Source: BMC Neurol. 2019 Dec 6;19:315. doi: 10.1186/s12883-019-1551-8 (PMC6898927; doi:10.1186/s12883-019-1551-8)
Supplement: Supplementary file 1 — Additional file 1: s1:Comparistions of patients' charactristics between outcomes (mRS 3-6 vs. mRS 0-2). s2:Comparistions of patients' charactristics between 90d alive vs. 90d death. s3:Comparistions of patients' charactristics between NO HT within 24h vs. HT within 24h. s4:Comparistions of patients' charactristics between poor clooateral vs. good collateral [file 12883_2019_1551_MOESM1_ESM.docx]

**Table S1**

|  | 90d mRS 3-6 | 90d mRS 0-2 | P-value |
| --- | --- | --- | --- |
| N | 119 | 68 |  |
| Age, mean (SD), years | 59.8 ( 9.9) | 59.0 (10.6) | 0.582 |
| SBP, mean (SD), mmHg | 158.6 (25.5) | 161.7 (25.3) | 0.417 |
| NIHSS score, median (IQR) | 29.0 (16.5-35.0) | 10.0 ( 4.8-20.0) | <0.001 |
| GCS score, median (IQR) | 6.0 (3.0-10.0) | 12.0 (8.8-15.0) | <0.001 |
| pc-ASPECTS on DWI, median (IQR) | 6.0 (5.0-8.0) | 7.0 (5.0-8.0) | 0.154 |
| PMI on DWI, median (IQR) | 3.0 (2.0-4.0) | 2.0 (0.0-2.0) | <0.001 |
| Onset to puncture time, median (IQR), hours | 7.5 (5.0-10.0) | 6.0 (4.8- 9.0) | 0.103 |
| Procedure time, median (IQR), hours | 2.0 (1.0-2.5) | 1.0 (1.0-2.0) | 0.005 |
| Onset to recanalization time, median (IQR), hours | 9.5 (7.0-12.0) | 8.0 (6.0-10.6) | 0.027 |
| Sex |  |  | 0.429 |
| Male | 98 (82.4%) | 59 (86.8%) |  |
| Female | 21 (17.6%) | 9 (13.2%) |  |
| Hypertension |  |  | 0.831 |
| No | 35 (29.4%) | 19 (27.9%) |  |
| Yes | 84 (70.6%) | 49 (72.1%) |  |
| Diabetes mellitus |  |  | 0.385 |
| No | 84 (70.6%) | 52 (76.5%) |  |
| Yes | 35 (29.4%) | 16 (23.5%) |  |
| Dyslipidemia |  |  | 0.386 |
| No | 102 (85.7%) | 55 (80.9%) |  |
| Yes | 17 (14.3%) | 13 (19.1%) |  |
| Coronary heart disease |  |  | 0.815 |
| No | 105 (88.2%) | 61 (89.7%) |  |
| Yes | 14 (11.8%) | 7 (10.3%) |  |
| Atrial fibrillation |  |  | 0.532 |
| No | 113 (95.0%) | 63 (92.6%) |  |
| Yes | 6 ( 5.0%) | 5 ( 7.4%) |  |
| Prior stroke |  |  | 0.862 |
| No | 95 (79.8%) | 55 (80.9%) |  |
| Yes | 24 (20.2%) | 13 (19.1%) |  |
| Premorbid mRS ≥ 3 |  |  | >0.999 |
| No | 113 (95.0%) | 65 (95.6%) |  |
| Yes | 6 ( 5.0%) | 3 ( 4.4%) |  |
| Current smoking |  |  | 0.265 |
| No | 78 (65.5%) | 39 (57.4%) |  |
| Yes | 41 (34.5%) | 29 (42.6%) |  |
| Occlusion site |  |  | 0.057 |
| Proximal BA (including intracranial VA) | 59 (49.6%) | 45 (66.2%) |  |
| Middle BA | 41 (34.5%) | 13 (19.1%) |  |
| Distal BA | 19 (16.0%) | 10 (14.7%) |  |
| Tandem lesion |  |  | 0.383 |
| No | 101 (84.9%) | 61 (89.7%) |  |
| Yes | 18 (15.1%) | 7 (10.3%) |  |
| Underlying ICAS |  |  | 0.886 |
| No | 45 (37.8%) | 25 (36.8%) |  |
| Yes | 74 (62.2%) | 43 (63.2%) |  |
| Presence of PcomA |  |  | 0.528 |
| No | 45 (37.8%) | 23 (33.8%) |  |
| Unilateral | 57 (47.9%) | 31 (45.6%) |  |
| Bilateral | 17 (14.3%) | 14 (20.6%) |  |
| ASITN/SIR collateral system |  |  | 0.018 |
| Grade 0-1 | 57 (47.9%) | 21 (30.9%) |  |
| Grade 2 | 53 (44.5%) | 34 (50.0%) |  |
| Grade 3-4 | 9 ( 7.6%) | 13 (19.1%) |  |
| Stroke subtype by TOAST criteria |  |  | 0.813 |
| Large artery arteriosclerosis | 97 (81.5%) | 54 (79.4%) |  |
| Cardioembolic | 17 (14.3%) | 12 (17.6%) |  |
| Other or unknown etiology | 5 ( 4.2%) | 2 ( 2.9%) |  |
| Prior use of intravenous tPA |  |  | 0.462 |
| No | 98 (82.4%) | 53 (77.9%) |  |
| Yes | 21 (17.6%) | 15 (22.1%) |  |
| General anaesthesia |  |  | <0.001 |
| No | 16 (13.4%) | 24 (35.3%) |  |
| Yes | 103 (86.6%) | 44 (64.7%) |  |
| Use of stent-retriever |  |  | 0.013 |
| No | 24 (20.2%) | 25 (36.8%) |  |
| Yes | 95 (79.8%) | 43 (63.2%) |  |
| Stent-retriever diameter |  |  | 0.403 |
| 4mm | 58 (61.1%) | 23 (53.5%) |  |
| 6mm | 37 (38.9%) | 20 (46.5%) |  |
| Stent-retriever length |  |  | 0.240 |
| 15-20mm | 65 (68.4%) | 25 (58.1%) |  |
| 30mm | 30 (31.6%) | 18 (41.9%) |  |
| No. of passes |  |  | 0.315 |
| ≤ 1 | 44 (46.3%) | 26 (60.5%) |  |
| 2 | 29 (30.5%) | 9 (20.9%) |  |
| ≥ 3 | 22 (23.2%) | 8 (18.6%) |  |
| Intra-arterial tPA or Urokinase |  |  | 0.818 |
| No | 91 (76.5%) | 53 (77.9%) |  |
| Yes | 28 (23.5%) | 15 (22.1%) |  |
| Balloon angioplasty |  |  | 0.003 |
| No | 45 (37.8%) | 41 (60.3%) |  |
| Yes | 74 (62.2%) | 27 (39.7%) |  |
| Stenting |  |  | 0.038 |
| No | 53 (44.5%) | 41 (60.3%) |  |
| Yes | 66 (55.5%) | 27 (39.7%) |  |

**Table S2**

|  | 90d alive | 90d death | P-value |
| --- | --- | --- | --- |
| N | 149 | 38 |  |
| Age, mean (SD), years | 59.7 (10.3) | 58.9 ( 9.6) | 0.663 |
| SBP, mean (SD), mmHg | 159.7 (23.9) | 159.8 (31.3) | 0.981 |
| NIHSS score, median (IQR) | 17.0( 9.0-31.0) | 34.0(29.0-35.0) | <0.001 |
| GCS score, median (IQR) | 10.0(5.0-13.0) | 3.0 (3.0- 6.0) | <0.001 |
| pc-ASPECTS on DWI, median (IQR) | 7.0 (5.0-8.0) | 6.0 (5.0-8.0) | 0.459 |
| PMI on DWI, median (IQR) | 2.0 (1.0-3.8) | 3.0 (2.0-4.0) | 0.278 |
| Onset to puncture time, median (IQR), hours | 7.0 (5.0- 9.1) | 8.0 (5.0-10.0) | 0.675 |
| Procedure time, median (IQR), hours | 1.5 (1.0-2.0) | 2.0 (1.8-3.4) | <0.001 |
| Onset to recanalization time, median (IQR), hours | 8.5 (6.0-11.0) | 9.0(7.0-14.0) | 0.133 |
| Sex |  |  | 0.332 |
| Male | 127 (85.2%) | 30 (78.9%) |  |
| Female | 22 (14.8%) | 8 (21.1%) |  |
| Hypertension |  |  | 0.991 |
| No | 43 (28.9%) | 11 (28.9%) |  |
| Yes | 106 (71.1%) | 27 (71.1%) |  |
| Diabetes mellitus |  |  | 0.059 |
| No | 113 (75.8%) | 23 (60.5%) |  |
| Yes | 36 (24.2%) | 15 (39.5%) |  |
| Dyslipidemia |  |  | >0.999 |
| No | 125 (83.9%) | 32 (84.2%) |  |
| Yes | 24 (16.1%) | 6 (15.8%) |  |
| Coronary heart disease |  |  | 0.386 |
| No | 134 (89.9%) | 32 (84.2%) |  |
| Yes | 15 (10.1%) | 6 (15.8%) |  |
| Atrial fibrillation |  |  | 0.467 |
| No | 139 (93.3%) | 37 (97.4%) |  |
| Yes | 10 ( 6.7%) | 1 ( 2.6%) |  |
| Prior stroke |  |  | 0.011 |
| No | 114 (76.5%) | 36 (94.7%) |  |
| Yes | 35 (23.5%) | 2 ( 5.3%) |  |
| Premorbid mRS ≥ 3 |  |  | >0.999 |
| No | 142 (95.3%) | 36 (94.7%) |  |
| Yes | 7 ( 4.7%) | 2 ( 5.3%) |  |
| Current smoking |  |  | 0.226 |
| No | 90 (60.4%) | 27 (71.1%) |  |
| Yes | 59 (39.6%) | 11 (28.9%) |  |
| Occlusion site |  |  | 0.536 |
| Proximal BA (including intracranial VA) | 85 (57.0%) | 19 (50.0%) |  |
| Middle BA | 43 (28.9%) | 11 (28.9%) |  |
| Distal BA | 21 (14.1%) | 8 (21.1%) |  |
| Tandem lesion |  |  | 0.006 |
| No | 135 (90.6%) | 27 (71.1%) |  |
| Yes | 14 ( 9.4%) | 11 (28.9%) |  |
| Underlying ICAS |  |  | 0.156 |
| No | 52 (34.9%) | 18 (47.4%) |  |
| Yes | 97 (65.1%) | 20 (52.6%) |  |
| Presence of PcomA |  |  | 0.464 |
| No | 57 (38.3%) | 11 (28.9%) |  |
| Unilateral | 69 (46.3%) | 19 (50.0%) |  |
| Bilateral | 23 (15.4%) | 8 (21.1%) |  |
| ASITN/SIR collateral system |  |  | 0.231 |
| Grade 0-1 | 58 (38.9%) | 20 (52.6%) |  |
| Grade 2 | 71 (47.7%) | 16 (42.1%) |  |
| Grade 3-4 | 20 (13.4%) | 2 ( 5.3%) |  |
| Stroke subtype by TOAST criteria |  |  | 0.743 |
| Large artery arteriosclerosis | 120 (80.5%) | 31 (81.6%) |  |
| Cardioembolic | 24 (16.1%) | 5 (13.2%) |  |
| Other or unknown etiology | 5 ( 3.4%) | 2 ( 5.3%) |  |
| Prior use of intravenous tPA |  |  | 0.649 |
| No | 119 (79.9%) | 32 (84.2%) |  |
| Yes | 30 (20.1%) | 6 (15.8%) |  |
| General anaesthesia |  |  | 0.078 |
| No | 36 (24.2%) | 4 (10.5%) |  |
| Yes | 113 (75.8%) | 34 (89.5%) |  |
| Use of stent-retriever |  |  | 0.147 |
| No | 43 (28.9%) | 6 (15.8%) |  |
| Yes | 106 (71.1%) | 32 (84.2%) |  |
| Stent-retriever diameter |  |  | 0.188 |
| 4mm | 59 (55.7%) | 22 (68.8%) |  |
| 6mm | 47 (44.3%) | 10 (31.2%) |  |
| Stent-retriever length |  |  | 0.080 |
| 15-20mm | 65 (61.3%) | 25 (78.1%) |  |
| 30mm | 41 (38.7%) | 7 (21.9%) |  |
| No. of passes |  |  | 0.034 |
| ≤ 1 | 59 (55.7%) | 11 (34.4%) |  |
| 2 | 29 (27.4%) | 9 (28.1%) |  |
| ≥ 3 | 18 (17.0%) | 12 (37.5%) |  |
| Intra-arterial tPA or Urokinase |  |  | 0.084 |
| No | 119 (79.9%) | 25 (65.8%) |  |
| Yes | 30 (20.1%) | 13 (34.2%) |  |
| Balloon angioplasty |  |  | 0.590 |
| No | 70 (47.0%) | 16 (42.1%) |  |
| Yes | 79 (53.0%) | 22 (57.9%) |  |
| Stenting |  |  | 0.971 |
| No | 75 (50.3%) | 19 (50.0%) |  |
| Yes | 74 (49.7%) | 19 (50.0%) |  |

**Table S3**

|  | No HT within 24h | HT within 24h | P-value |
| --- | --- | --- | --- |
| N | 158 | 29 |  |
| Age, mean (SD), years | 59.3 (10.2) | 60.5 (10.1) | 0.578 |
| SBP, mean (SD), mmHg | 159.7 (25.0) | 159.8 (28.0) | 0.980 |
| NIHSS score, median (IQR) | 20.0 (10.0-34.0) | 31.0 (16.0-35.0) | 0.036 |
| GCS score, median (IQR) | 9.0 (4.2-13.0) | 5.0 (3.0-10.0) | 0.029 |
| pc-ASPECTS on DWI, median (IQR) | 6.0 (5.0-8.0) | 6.0 (4.2-8.0) | 0.741 |
| PMI on DWI, median (IQR) | 2.0 (1.0-4.0) | 2.0 (0.0-4.0) | 0.953 |
| Onset to puncture time, median (IQR), hours | 7.0 (5.0-10.0) | 8.0 (5.0- 9.5) | 0.410 |
| Procedure time, median (IQR), hours | 1.5 (1.0-2.0) | 2.0 (1.0-2.8) | 0.243 |
| Onset to recanalization time, median (IQR), hours | 8.8 (6.5-11.6) | 10.0 (7.0-12.6) | 0.373 |
| Sex |  |  | 0.581 |
| Male | 131 (82.9%) | 26 (89.7%) |  |
| Female | 27 (17.1%) | 3 (10.3%) |  |
| Hypertension |  |  | >0.999 |
| No | 46 (29.1%) | 8 (27.6%) |  |
| Yes | 112 (70.9%) | 21 (72.4%) |  |
| Diabetes mellitus |  |  | 0.368 |
| No | 117 (74.1%) | 19 (65.5%) |  |
| Yes | 41 (25.9%) | 10 (34.5%) |  |
| Dyslipidemia |  |  | 0.581 |
| No | 131 (82.9%) | 26 (89.7%) |  |
| Yes | 27 (17.1%) | 3 (10.3%) |  |
| Coronary heart disease |  |  | >0.999 |
| No | 140 (88.6%) | 26 (89.7%) |  |
| Yes | 18 (11.4%) | 3 (10.3%) |  |
| Atrial fibrillation |  |  | >0.999 |
| No | 148 (93.7%) | 28 (96.6%) |  |
| Yes | 10 ( 6.3%) | 1 ( 3.4%) |  |
| Prior stroke |  |  | 0.805 |
| No | 126 (79.7%) | 24 (82.8%) |  |
| Yes | 32 (20.3%) | 5 (17.2%) |  |
| Premorbid mRS ≥ 3 |  |  | >0.999 |
| No | 150 (94.9%) | 28 (96.6%) |  |
| Yes | 8 ( 5.1%) | 1 ( 3.4%) |  |
| Current smoking |  |  | 0.108 |
| No | 95 (60.1%) | 22 (75.9%) |  |
| Yes | 63 (39.9%) | 7 (24.1%) |  |
| Occlusion site |  |  | 0.851 |
| Proximal BA (including intracranial VA) | 87 (55.1%) | 17 (58.6%) |  |
| Middle BA | 47 (29.7%) | 7 (24.1%) |  |
| Distal BA | 24 (15.2%) | 5 (17.2%) |  |
| Tandem lesion |  |  | >0.999 |
| No | 137 (86.7%) | 25 (86.2%) |  |
| Yes | 21 (13.3%) | 4 (13.8%) |  |
| Underlying ICAS |  |  | 0.371 |
| No | 57 (36.1%) | 13 (44.8%) |  |
| Yes | 101 (63.9%) | 16 (55.2%) |  |
| Presence of PcomA |  |  | 0.139 |
| No | 55 (34.8%) | 13 (44.8%) |  |
| Unilateral | 79 (50.0%) | 9 (31.0%) |  |
| Bilateral | 24 (15.2%) | 7 (24.1%) |  |
| ASITN/SIR collateral system |  |  | 0.778 |
| Grade 0-1 | 64 (40.5%) | 14 (48.3%) |  |
| Grade 2 | 75 (47.5%) | 12 (41.4%) |  |
| Grade 3-4 | 19 (12.0%) | 3 (10.3%) |  |
| Stroke subtype by TOAST criteria |  |  | 0.635 |
| Large artery arteriosclerosis | 129 (81.6%) | 22 (75.9%) |  |
| Cardioembolic | 23 (14.6%) | 6 (20.7%) |  |
| Other or unknown etiology | 6 ( 3.8%) | 1 ( 3.4%) |  |
| Prior use of intravenous tPA |  |  | 0.451 |
| No | 129 (81.6%) | 22 (75.9%) |  |
| Yes | 29 (18.4%) | 7 (24.1%) |  |
| General anaesthesia |  |  | >0.999 |
| No | 34 (21.5%) | 6 (20.7%) |  |
| Yes | 124 (78.5%) | 23 (79.3%) |  |
| Use of stent-retriever |  |  | 0.646 |
| No | 43 (27.2%) | 6 (20.7%) |  |
| Yes | 115 (72.8%) | 23 (79.3%) |  |
| Stent-retriever diameter |  |  | 0.821 |
| 4mm | 68 (59.1%) | 13 (56.5%) |  |
| 6mm | 47 (40.9%) | 10 (43.5%) |  |
| Stent-retriever length |  |  | 0.347 |
| 15-20mm | 77 (67.0%) | 13 (56.5%) |  |
| 30mm | 38 (33.0%) | 10 (43.5%) |  |
| No. of passes |  |  | 0.213 |
| ≤ 1 | 62 (53.9%) | 8 (34.8%) |  |
| 2 | 29 (25.2%) | 9 (39.1%) |  |
| ≥ 3 | 24 (20.9%) | 6 (26.1%) |  |
| Intra-arterial tPA or Urokinase |  |  | 0.148 |
| No | 125 (79.1%) | 19 (65.5%) |  |
| Yes | 33 (20.9%) | 10 (34.5%) |  |
| Balloon angioplasty |  |  | 0.059 |
| No | 68 (43.0%) | 18 (62.1%) |  |
| Yes | 90 (57.0%) | 11 (37.9%) |  |
| Stenting |  |  | 0.009 |
| No | 73 (46.2%) | 21 (72.4%) |  |
| Yes | 85 (53.8%) | 8 (27.6%) |  |

**Table S4**

|  | Poor collateral(ASITN/SIR 0-1) | Good collateral(ASITN/SIR 2-4) | P-value |
| --- | --- | --- | --- |
| N | 78 | 109 |  |
| Age, mean (SD), years | 60.8 ( 9.1) | 58.6 (10.8) | 0.137 |
| SBP, mean (SD), mmHg | 160.4 (26.7) | 159.3 (24.7) | 0.773 |
| NIHSS score, median (IQR) | 29.0 (15.2-35.0) | 17.0 ( 8.0-31.0) | 0.001 |
| GCS score, median (IQR) | 6.0 (3.0-10.0) | 10.0 (5.0-14.0) | 0.002 |
| pc-ASPECTS on DWI, median (IQR) | 7.0 (5.0-8.0) | 6.0 (5.0-8.0) | 0.955 |
| PMI on DWI, median (IQR) | 2.0 (0.0-4.0) | 2.0 (1.0-3.8) | 0.734 |
| Onset to puncture time, median (IQR), hours | 7.0 (5.0- 9.0) | 7.0 (5.0-10.0) | 0.968 |
| Procedure time, median (IQR), hours | 2.0 (1.0-2.4) | 1.5 (1.0-2.0) | 0.055 |
| Onset to recanalization time, median (IQR), hours | 8.8 (7.0-12.0) | 9.0 (6.0-11.5) | 0.579 |
| Sex |  |  | 0.315 |
| Male | 63 (80.8%) | 94 (86.2%) |  |
| Female | 15 (19.2%) | 15 (13.8%) |  |
| Hypertension |  |  | 0.409 |
| No | 20 (25.6%) | 34 (31.2%) |  |
| Yes | 58 (74.4%) | 75 (68.8%) |  |
| Diabetes mellitus |  |  | 0.364 |
| No | 54 (69.2%) | 82 (75.2%) |  |
| Yes | 24 (30.8%) | 27 (24.8%) |  |
| Dyslipidemia |  |  | 0.156 |
| No | 69 (88.5%) | 88 (80.7%) |  |
| Yes | 9 (11.5%) | 21 (19.3%) |  |
| Coronary heart disease |  |  | 0.244 |
| No | 72 (92.3%) | 94 (86.2%) |  |
| Yes | 6 ( 7.7%) | 15 (13.8%) |  |
| Atrial fibrillation |  |  | 0.530 |
| No | 72 (92.3%) | 104 (95.4%) |  |
| Yes | 6 ( 7.7%) | 5 ( 4.6%) |  |
| Prior stroke |  |  | 0.872 |
| No | 63 (80.8%) | 87 (79.8%) |  |
| Yes | 15 (19.2%) | 22 (20.2%) |  |
| Premorbid mRS ≥ 3 |  |  | 0.309 |
| No | 76 (97.4%) | 102 (93.6%) |  |
| Yes | 2 ( 2.6%) | 7 ( 6.4%) |  |
| Current smoking |  |  | 0.714 |
| No | 50 (64.1%) | 67 (61.5%) |  |
| Yes | 28 (35.9%) | 42 (38.5%) |  |
| Occlusion site |  |  | 0.187 |
| Proximal BA (including intracranial VA) | 47 (60.3%) | 57 (52.3%) |  |
| Middle BA | 17 (21.8%) | 37 (33.9%) |  |
| Distal BA | 14 (17.9%) | 15 (13.8%) |  |
| Tandem lesion |  |  | 0.120 |
| No | 64 (82.1%) | 98 (89.9%) |  |
| Yes | 14 (17.9%) | 11 (10.1%) |  |
| Underlying ICAS |  |  | 0.581 |
| No | 31 (39.7%) | 39 (35.8%) |  |
| Yes | 47 (60.3%) | 70 (64.2%) |  |
| Presence of PcomA |  |  | <0.001 |
| No | 45 (57.7%) | 23 (21.1%) |  |
| Unilateral | 28 (35.9%) | 60 (55.0%) |  |
| Bilateral | 5 ( 6.4%) | 26 (23.9%) |  |
| Stroke subtype by TOAST criteria |  |  | 0.387 |
| Large artery arteriosclerosis | 64 (82.1%) | 87 (79.8%) |  |
| Cardioembolic | 13 (16.7%) | 16 (14.7%) |  |
| Other or unknown etiology | 1 ( 1.3%) | 6 ( 5.5%) |  |
| Prior use of intravenous tPA |  |  | 0.448 |
| No | 65 (83.3%) | 86 (78.9%) |  |
| Yes | 13 (16.7%) | 23 (21.1%) |  |
| General anaesthesia |  |  | 0.542 |
| No | 15 (19.2%) | 25 (22.9%) |  |
| Yes | 63 (80.8%) | 84 (77.1%) |  |
| Use of stent-retriever |  |  | 0.134 |
| No | 16 (20.5%) | 33 (30.3%) |  |
| Yes | 62 (79.5%) | 76 (69.7%) |  |
| No. of passes |  |  | 0.577 |
| ≤ 1 | 30 (48.4%) | 40 (52.6%) |  |
| 2 | 16 (25.8%) | 22 (28.9%) |  |
| ≥ 3 | 16 (25.8%) | 14 (18.4%) |  |
| Intra-arterial tPA or Urokinase |  |  | 0.708 |
| No | 59 (75.6%) | 85 (78.0%) |  |
| Yes | 19 (24.4%) | 24 (22.0%) |  |
| Balloon angioplasty |  |  | 0.527 |
| No | 38 (48.7%) | 48 (44.0%) |  |
| Yes | 40 (51.3%) | 61 (56.0%) |  |
| Stenting |  |  | 0.341 |
| No | 36 (46.2%) | 58 (53.2%) |  |
| Yes | 42 (53.8%) | 51 (46.8%) |  |
